# The accuracy of standard enthalpies and entropies for phases of petrological interest derived from density-functional calculations

**DOI:** 10.1007/s00410-018-1514-x

**Published:** 2018-10-16

**Authors:** Artur Benisek, Edgar Dachs

**Affiliations:** Chemistry and Physics of Materials, University of Salzburg, Jakob-Haringerstr. 2a, 5020 Salzburg, Austria

**Keywords:** Thermodynamics, First principles, Ab initio, DFT, CASTEP, Feldspars, Aluminosilicates, Olivines, Pyroxenes, Garnets, Micas, Amphiboles, Perovskite

## Abstract

The internal energies and entropies of 21 well-known minerals were calculated using the density functional theory (DFT), viz. kyanite, sillimanite, andalusite, albite, microcline, forsterite, fayalite, diopside, jadeite, hedenbergite, pyrope, grossular, talc, pyrophyllite, phlogopite, annite, muscovite, brucite, portlandite, tremolite, and CaTiO_3_–perovskite. These thermodynamic quantities were then transformed into standard enthalpies of formation from the elements and standard entropies enabling a direct comparison with tabulated values. The deviations from reference enthalpy and entropy values are in the order of several kJ/mol and several J/mol/K, respectively, from which the former is more relevant. In the case of phase transitions, the DFT-computed thermodynamic data of involved phases turned out to be accurate and using them in phase diagram calculations yields reasonable results. This is shown for the Al_2_SiO_5_ polymorphs. The DFT-based phase boundaries are comparable to those derived from internally consistent thermodynamic data sets. They even suggest an improvement, because they agree with petrological observations concerning the coexistence of kyanite + quartz + corundum in high-grade metamorphic rocks, which are not reproduced correctly using internally consistent data sets. The DFT-derived thermodynamic data are also accurate enough for computing the *P–T* positions of reactions that are characterized by relatively large reaction enthalpies (> 100 kJ/mol), i.e., dehydration reactions. For reactions with small reaction enthalpies (a few kJ/mol), the DFT errors are too large. They, however, are still far better than enthalpy and entropy values obtained from estimation methods.

## Introduction

Investigating the standard enthalpy of formation from the elements (Δ_f_*H*^298.15^) and the standard entropy (*S*^298.15^) of mineral end members of geological and cosmochemical relevance is a prerequisite for reliable phase diagram calculations and still needed in many aspects: first, because such standard data of some mineral end members are derived from a limited number of experiments and largely missing for chemically more complex systems (e.g., Ti-containing end members of many solid solutions such as pyroxenes, micas, amphiboles) and, second, because there are important mineral solid solutions whose thermodynamic description needs data from end members which do not exist physically at all (e.g., Al-rich biotite end members). The experimental data basis is also poor for rare parageneses (e.g., minerals in calcium–aluminium-rich inclusions of primitive meteorites). This has the consequence that thermodynamic calculations including such end members suffer from missing accuracy.

Nowadays, the generation of such end member thermodynamic data is possible in a relatively short time by using the density functional theory (DFT). These data can then be integrated into existing thermodynamic data sets making them available to a broad range of applications.

The following question, however, arise: Are the DFT results accurate enough?

The uncertainties in Δ_f_*H*^298.15^ given in internally consistent thermodynamic data sets are in the order of a few kJ/mol for well-known phases. Because Δ_f_*H*^298.15^ data of such mineral end members are based on a large number of experimental data, it can be assumed that their accuracy is of the same order. The uncertainties of reaction enthalpies derived from solution calorimetry is around a few kJ/mol (e.g., Majzlan et al. 2017), and those of standard entropy values derived from relaxation calorimetry is in the order of a few J/mol/K and does not exceed 1% (Dachs and Benisek 2011). The question of the required accuracy of DFT-calculated standard data needed for reliable phase diagram calculations, is, therefore, easy to answer. It should not be much larger than the uncertainties of the two calorimetric methods mentioned, allowing incorporating them in the development of internally consistent databases. The quality of DFT-calculated thermodynamic data should therefore be tested by comparison with those from well-known mineral end members with their high accuracy.

The comparison of DFT-calculated energies to enthalpies listed in thermodynamic data sets is, however, directly not possible, first, because their zero levels are different. In thermodynamic data sets, the enthalpies of the elements are set to zero, whereas in DFT calculations, the atom cores separated from the valence electrons have zero potential. The most obvious way for solving this problem is to investigate the DFT energies of the minerals in question as well as of their constituting elements and to calculate then the formation energies from the elements. Since the elements and most mineral end members belong to quite different classes of materials, this approach leads to large errors (Benisek and Dachs 2017). A more appropriate approach is to calculate the energy of the phases under consideration, as well as of the binary oxides they are made of (Hautier et al. 2012). This enables the determination of the formation energy of a phase from its constituting oxides. Adding Δ_f_*H* values of the oxides, which are tabulated with high accuracy in the Janaf-tables (Chase 1998), the Δ_f_*H* value of a phase from the elements can be determined. A second problem for the comparison of tabulated and DFT-based Δ_f_*H* values is the fact that energies calculated by the DFT method are internal energies at 0 K, which need to be transformed into enthalpies at 298.15 K.

The standard entropy of a phase can also be calculated by the DFT method by investigating the lattice dynamics, which results in heat capacity data. This heat capacity, however, is that at constant volume (*C*_V_) and the entropy derived from it is not directly comparable to *S*^298.15^ of thermodynamic data sets, which is based on the heat capacity at constant pressure (*C*_P_). Principally, *C*_P_ could be computed by the DFT method by using the quasiharmonic approximation (Born and Huang 1956), which is, however, a time-consuming method. If this method must be avoided, *C*_V_ has to be transformed into *C*_P_ somehow different to make the entropies comparable.

Since the use of the quasiharmonic approximation is found to be too time-consuming for a broad study on the accuracy of DFT-calculated enthalpies and entropies, we offer in this publication an efficient method to transform these quantities, enabling a direct comparison between DFT-calculated and tabulated properties on a large number of minerals. Such a comparison on many well-known mineral end members is needed to answer the question, if DFT-calculated thermodynamic properties are able to complement calorimetric experiments. The errors generated by the transformation method should be much smaller than those generated by the DFT method itself to make a reliable comparison possible.

In this study, we present DFT-based Δ_f_*H*^298.15^ and *S*^298.15^ values of 21 well-known mineral end members. These data were applied to phase equilibrium calculations to assess the associated uncertainties and to discuss relevant petrological problems.

## Experimental methods

### Computational methods

Quantum–mechanical calculations were based on the DFT plane wave pseudopotential approach implemented in the CASTEP code (Clark et al. 2005) included in the Materials Studio software from Accelrys^®^. The calculations used the local density approximation (LDA) for the exchange–correlation functional (Ceperley and Alder 1980) and norm-conserving pseudopotentials to describe the core–valence interactions. For the k-point sampling of the investigated unit cells, a Monkhorst–Pack grid (spacing of 0.02/Å) was used (Monkhorst and Pack 1976) and convergence was tested by performing calculations using a denser k-point grid. The structural relaxation was calculated applying the BFGS algorithm, where the threshold for the force on the atom was 0.01 eV/Å. The calculations on Fe-containing minerals used an ultrasoft pseudopotential and the LDA + *U* approach (with *U* = 4.0 eV applied to Fe *d* orbitals). In addition to the LDA calculations, the gradient-corrected functional (GGA-PBE) from Perdew et al. (1996), its revised form for solids (GGA-PBESOL, Perdew et al. 2008), and hybrid functionals such as B3LYP (Becke 1993) and PBE0 (Adamo 1999) were used for comparison reasons in some cases. Using the hybrid functionals, the single point energies were calculated on a LDA-geometry optimized cell.

The lattice dynamical calculations were performed for the relaxed structures within the linear response approximation implemented in CASTEP using the interpolation approach and a wider k-point grid (spacing of 0.05/Å) compared to the energy calculations. The lattice dynamical calculations on Fe-containing minerals were based on the finite displacement approach, which calculated the forces on perturbed configurations in a super cell with positive and negative displacements.

### Transformation of DFT-calculated quantities to those tabulated in thermodynamic data sets

#### Heat capacity

The difference between the heat capacity at constant volume (*C*_V_) and that at constant pressure (*C*_P_) is given by the following equation (e.g., Cemic 2005):

1 $${C_{\text{P}}}\; - \;{C_{\text{V}}}\;=\;{\alpha ^{\text{2}}}{K_{\text{T}}}VT,$$

where *V* denotes the molar volume, *α* the thermal expansion coefficient, and *K*_T_ the isothermal bulk modulus. To make the transformation from *C*_V_ to *C*_P_, the values for *V, α*, and *K*_T_ are assumed to be similar within a given mineral group (e.g., pyroxenes, micas) so that the relative difference between *C*_V_ and *C*_P_

2 $$\Delta {C^{{\text{rel}}}}\;=\;\left( {{C_{\text{P}}}\; - \;{C_{\text{V}}}} \right)/{C_{\text{P}}},$$

can be averaged for a particular mineral group. This enables that DFT-calculated *C*_V_ of a phase belonging to this family can be transformed to *C*_P_.

#### Enthalpy and entropy

The DFT method was used to compute the internal reaction energy at 0 K (Δ_R_*U*^0K^) of the formation of a mineral phase from its oxides. Based on the generalized reaction,

3 $${\text{2 AO}}\;+\;{\text{B}}{{\text{O}}_{\text{2}}}\;=\;{{\text{A}}_{\text{2}}}{\text{B}}{{\text{O}}_{\text{4}}},$$

Δ_R_*U*^0K^ is given by:

4 $${\Delta _{\text{R}}}{U^{0{\text{K}}}}\;=\;{U^{0{\text{K}}}}_{{{{\text{A}}_{\text{2}}}{\text{B}}{{\text{O}}_{\text{4}}}}}\; - \;\left( {{\text{2}}*{U^{0{\text{K}}}}_{{{\text{AO}}}}+{U^{0{\text{K}}}}_{{{\text{B}}{{\text{O}}_{\text{2}}}}}} \right),$$

where *U*^0K^_AO_, ${U^{0{\text{K}}}}_{{{\text{B}}{{\text{O}}_{\text{2}}}}}$, and ${U^{0{\text{K}}}}_{{{{\text{A}}_{\text{2}}}{\text{B}}{{\text{O}}_{\text{4}}}}}$ are the internal energies of the reactants at 0 K.

As a next step, it was assumed that Δ_R_*U*^0K^ of reaction (3) was equal to the reaction enthalpy at 0 K (Δ_R_*H*^0K^), i.e.,

5 $${\Delta _{\text{R}}}{U^{0{\text{K}}}} \approx {\Delta _{\text{R}}}{H^{0{\text{K}}}}.$$

Since Δ_R_*U*^0K^ is the reaction energy of solids at 0 K, this simplification does not lead to relevant errors because the volume term (*P*Δ_R_*V*) relating internal energy and enthalpy is very small and can be neglected (Hautier et al. 2012). The enthalpy of formation of A_2_BO_4_ from the elements at 0 K $\left( {{\Delta _{\text{f}}}{H^{0{\text{K}}}}_{{{{\text{A}}_{\text{2}}}{\text{B}}{{\text{O}}_{\text{4}}}}}} \right)$ was then calculated using accurate Δ_f_*H*^0K^ values of the constituting oxides taken from the Janaf-tables (Chase 1998) according to:

6 $${\Delta _{\text{f}}}{H^{0{\text{K}}}}_{{{{\text{A}}_{\text{2}}}{\text{B}}{{\text{O}}_{\text{4}}}}}~\;=\;{\Delta _{\text{R}}}{H^{0{\text{K}}}}\;+\;{\text{ 2}}*{\Delta _{\text{f}}}{H^{0{\text{K}}}}_{{{\text{AO}}}}+{\Delta _{\text{f}}}{H^{0{\text{K}}}}_{{{\text{B}}{{\text{O}}_{\text{2}}}}}.$$

The so-derived values contain only simplifications inherent in the DFT calculations. Using the above described approach to convert *C*_V_ to *C*_P_ (Sect. “Heat capacity”), the standard enthalpy was then computed via

7 $${\Delta _{\text{f}}}{H^{{\text{298}}.{\text{15}}}}={\Delta _{\text{f}}}{H^{0{\text{K}}}}+\int {{C_{\text{P}}}{\text{d}}T} .$$

**Fig. 1:**
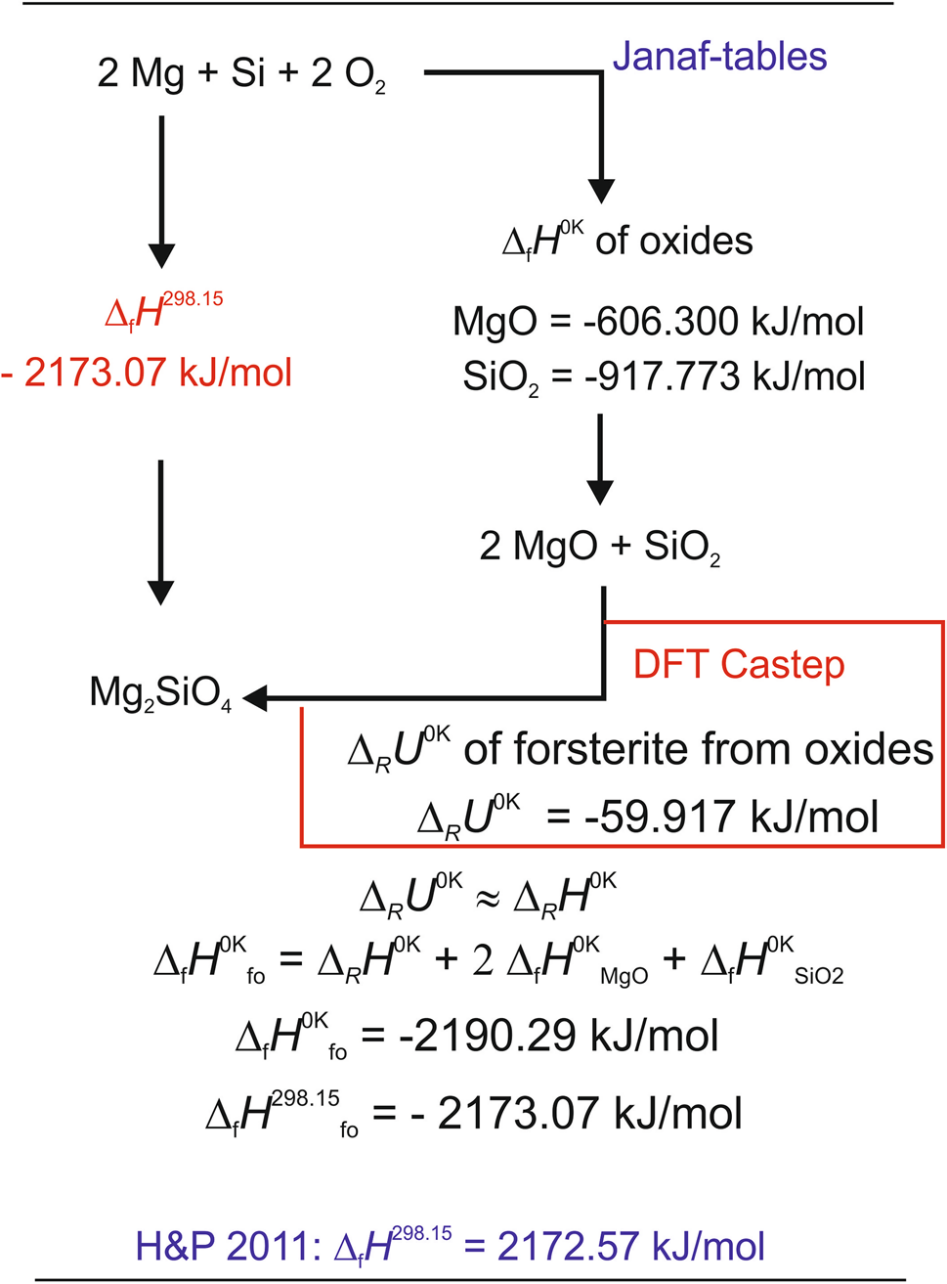
Sketch of the calculation procedure for forsterite as an example. The following reaction was investigated: 2MgO + SiO_2_ = Mg_2_SiO_4_. Its reaction energy at 0 K (Δ_R_*U*^0K^) was calculated by the DFT method and it is assumed that the reaction enthalpy at 0 K (Δ_R_*H*^0K^) is identical. Adding the formation enthalpies of the oxides at 0 K (Δ_f_*H*^0K^_ox_) to this Δ_R_*H*^0K^ value results in the formation enthalpy of forsterite from the elements at 0 K (Δ_f_*H*^0K^_fo_). The heat content from 0 to 298.15 K (= ∫*C*_P_^fo^ d*T*) is than added to this value yielding finally the standard enthalpy of formation of forsterite from the elements (Δ_f_*H*^298.15^_fo_)

A sketch of the complete calculation procedure is shown in Fig. 1 for forsterite as an example. The entropy was calculated by integrating the calculated *C*_P_/*T* values over the temperature range from 0 to 298.15 K, i.e.,

8 $${S^{298.15}}=\int {{C_{\text{P}}}/T{\text{d}}T} .$$

#### Fe-bearing minerals

For Fe-containing minerals, another transformation method was used to bypass problems caused by the magnetic phase transitions appearing in these systems, since the magnetic entropy cannot be determined directly with DFT (Hickel et al. 2011). The DFT-calculated reaction energies and entropies from the oxides (Eq. 3) at 0 K were transformed to 298.15 K using the calculated reaction heat capacities (Δ_R_*C*_V_) of Eq. (3). As shown later, the integrals of Δ_R_*C*_V_ and Δ_R_*C*_V_/*T* over temperature have similar values when compared to those of measured Δ_R_*C*_P_ and Δ_R_*C*_P_/*T*. This is because the effect of magnetic transformations on the heat capacity are present in both, the right and the left hand side of Eq. (3), thus partly eliminating their magnetic contribution. The same is true with the difference between heat capacity at constant volume and that at constant pressure. The reaction enthalpies and entropies at 298.15 K of mineral end members from their oxides were calculated according to

9 $${\Delta _{\text{R}}}{H^{{\text{298}}.{\text{15}}}} \approx {\Delta _{\text{R}}}{H^{0{\text{K}}}}~+\int {{\Delta _{\text{R}}}{C_{\text{V}}}{\text{d}}T} ,$$

10 $${\Delta _{\text{R}}}{S^{{\text{298}}.{\text{15}}}} \approx {\Delta _{\text{R}}}{S^{0{\text{K}}}}+\int {{\Delta _{\text{R}}}{C_{\text{V}}}/T{\text{d}}T} .$$

Finally, Δ_f_*H*^298.15^ and *S*^298.15^ of Fe-containing minerals were then calculated using the oxide data at 298.15 K from the Janaf-tables (Chase 1998).

#### OH-containing minerals

Several minerals containing OH groups were investigated. Consequently, reaction (3) has H_2_O on its left hand side. A directly calculated internal energy of H_2_O at 0 K using the DFT method showed, however, a mean systematic error of 38 kJ/mol. The DFT-based internal energy of H_2_O at 0 K was, therefore, shifted by this value improving the Δ_f_*H*^298.15^ values of the investigated OH-containing mineral end members.

### Investigated mineral end members and their structural models

The investigated minerals are listed with formulae and abbreviations in Table 1. The atomic distributions are fully ordered for most of the investigated minerals and there is no configurational contribution to the entropy for these cases. However, biotite end members and muscovite are characterized by a disordered distribution of Al and Si on tetrahedral sites. For these minerals, we investigated a larger number of cells having all possible configurations except the ones, where Al–O–Al bonds are present. This Al-avoidance treatment is in accordance with numerous studies (e.g., Circone et al. 1991; Palin et al. 2001; Vinograd and Putnis 1998), which all found that adjacent Al tetrahedra in micas are virtually absent. From the DFT results of all investigated cells, the mean values of *C*_P_ and Δ_f_*H*^298.15^ were calculated, which assumes that all investigated configurations are present and equally probable. The impact of this assumption on the accuracy of Δ_f_*H*^298.15^ and *S*^298.15^ was estimated to be in the order of kJ/mol and J/mol/K, respectively. The configurational entropy was calculated with a Al-avoidance model (e.g., Holland and Powell 1998) and was added to the vibrational entropy calculated by the DFT method.

**Table 1:** Formulae and abbreviations of the minerals used in this study

Group	Mineral	Abbreviation	Formula
Aluminosilicates	Andalusite	and	Al_2_SiO_5_
	Sillimanite	sill	Al_2_SiO_5_
	Kyanite	ky	Al_2_SiO_5_
Feldspars	Microcline	mic	KAlSi_3_O_8_
	Albite (low)	ab	NaAlSi_3_O_8_
Olivines	Forsterite	fo	Mg_2_SiO_4_
	Fayalite	fa	Fe_2_SiO_4_
Pyroxenes	Diopside	di	CaMgSi_2_O_6_
	Jadeite	jd	NaAlSi_2_O_6_
	Hedenbergite	hed	CaFeSi_2_O_6_
Garnets	Pyrope	py	Mg_3_Al_2_Si_3_O_12_
	Grossular	gr	Ca_3_Al_2_Si_3_O_12_
OH-containing minerals Micas Amphibole	Talc	ta	Mg_3_[(OH)_2_Si_4_O_10_]
	Pyrophyllite	pyp	Al_2_[(OH)_2_Si_4_O_10_]
	Phlogopite	phl	KMg_3_[(OH)_2_AlSi_3_O_10_]
	Annite	ann	KFe_3_[(OH)_2_AlSi_3_O_10_]
	Muscovite	mu	KAl_2_[(OH)_2_AlSi_3_O_10_]
	Brucite	bru	Mg(OH)_2_
	Portlandite	por	Ca(OH)_2_
	Tremolite	tr	Ca_2_Mg_5_[(OH)_2_Si_8_O_22_]
Oxide	CaTi-Perovskite	per	CaTiO_3_

## Results

### Heat capacity

**Fig. 2:**
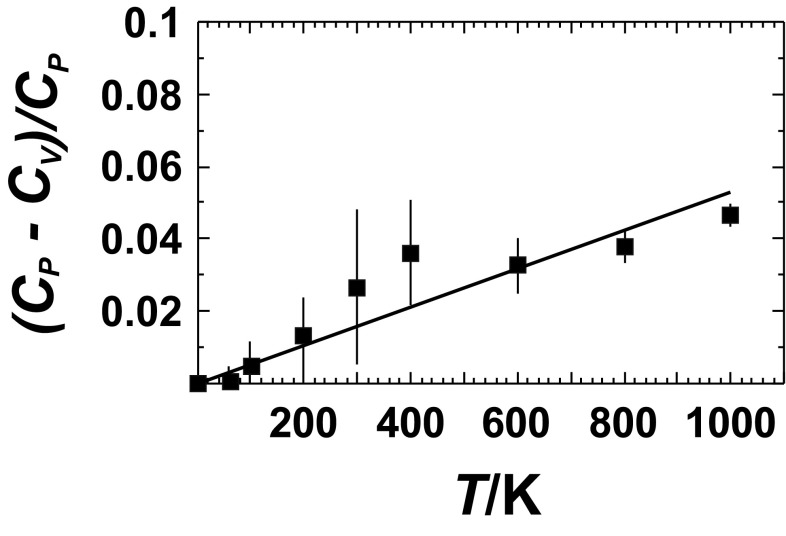
Relative mean difference between heat capacity at constant pressure (*C*_P_) and that at constant volume (*C*_V_) plotted as a function of temperature for the pyroxenes diopside and jadeite. Error bars represent 1 sd

The relative difference between *C*_V_ and *C*_P_ (Eq. 2) is shown in Fig. 2 for the pyroxenes as an example. The *C*_P_ data were taken from the literature, and *C*_V_ was calculated by the DFT method showing a linear relationship of Δ*C*^rel^ versus *T* (solid line in Fig. 2). The DFT-calculated *C*_V_ of the pyroxenes can thus easily be transformed to *C*_P_. This procedure was performed for the other mineral groups as well. The heat capacities of all investigated minerals are compared to reference values in Table 2 showing that deviations are in the order of a few J/mol/K. The impact of the adopted *C*_V_−*C*_P_ conversion on Δ_f_*H*^298.15^ and *S*^298.15^ is in the order of 0.1 kJ/mol and 1 J/mol/K, respectively, and is shown for Δ_f_*H*^298.15^ of low microcline in the next chapter. Using this procedure, DFT-calculated *C*_V_ values of unknown members of the mineral groups studied can be transformed to the corresponding *C*_P_ values.

**Table 2:** DFT-calculated heat capacity (*C*_P_) of Fe-free minerals

	*C*_P_/(J/mol/K) at various T/K				
	298.15	400	600	800	1000
and	120.3 (− 2.3)	147.0 (− 2.4)	175.6 (0.0)	190.4 (1.4)	198.7 (1.6)
sill	120.8 (− 2.9)	146.4 (− 2.5)	174.6 (0.4)	189.6 (1.9)	198.2 (2.2)
ky	122.0 (0.4)	149.2 (− 0.1)	177.4 (1.5)	191.7 (2.2)	199.7 (1.8)
mic	204.8 (0.4)	240.2 (0.4)	281.8 (4.0)	304.5 (5.8)	317.5 (5.5)
ab	203.4 (− 1.3)	239.4 (− 1.6)	281.5 (2.5)	304.3 (4.8)	317.5 (5.1)
fo	118.1 (− 0.5)	138.1 (1.5)	158.0 (1.8)	167.6 (0.2)	172.8 (− 2.2)
di	167.3 (0.7)	195.5 (− 0.9)	225.4 (0.9)	240.9 (2.1)	249.9 (2.0)
jd	162.2 (− 2.0)	191.4 (1.4)	222.8 (5.3)	239.3 (6.2)	248.8 (5.0)
py	326.9 (1.7)	385.5 (0.3)	445.0 (2.1)	474.1 (1.3)	489.5 (− 2.4)
gr	331.3 (2.1)	390.5 (0.7)	448.7 (2.2)	476.6 (1.1)	491.2 (− 2.4)
ta	325.6 (2.0)	386.0 (− 0.5)	449.8 (5.2)	483.0 (9.2)	502.2 (10.2)
pyp	294.3 (0.5)	353.9 (3.6)	419.3 (3.9)	453.9 (2.2)	474.0 (− 0.1)
phl	352.1 (− 2.7)	412.9 (− 1.5)	476.6 (1.6)	509.7 (3.5)	528.9 (4.4)
mu	319.2 (− 2.7)	379.8 (− 6.8)	444.8 (− 8.7)	479.7 (− 10.7)	500.0 (− 13.7)
bru	76.6 (− 0.6)	90.3 (− 1.4)	106.9 (2.4)	119.1 (8.6)	129.9 (15.0)
por	82.7 (− 4.8)	94.1 (− 4.3)	107.7 (0.2)	118.2 (4.7)	127.9 (9.9)
tr	657.9 (2.4)	773.0 (− 5.2)	894.0 (− 0.4)	955.7 (1.6)	990.2 (− 1.9)
per	98.5 (0.8)	110.1 (− 4.1)	121.2 (− 1.4)	127.0 (1.2)	130.7 (1.5)

Numbers in brackets give the deviations (*C*_P_^calc^−*C*_P_^ref^) from reference values (Holland and Powell 2011)

As already mentioned in Sect. “Fe-bearing minerals’’, Fe-containing minerals were calculated using a different routine. In Table 3, the integrals of the calculated Δ_R_*C*_V_ and Δ_R_*C*_V_/*T* over temperature are compared to those of measured Δ_R_*C*_P_ and Δ_R_*C*_P_/*T*, showing acceptable agreement. The impact of the simplifications adopted in this procedure on the accuracy of Δ_f_*H*^298.15^ and *S*^298.15^ were estimated to be in the order of 1 kJ/mol for the enthalpy and some J/mol/K for the entropy.

**Table 3:** Enthalpy and entropy change due to heating from 0 to 298.15 K for the investigated Fe-containing minerals, i.e., hedenbergite (hed), fayalite (fa), and annite (ann)

	Heat content		Entropy	
	Calorimetry	DFT	Calorimetry	DFT
	$\mathop \int \limits_{0}^{{298.15}} {C_{\text{P}}}{\text{d}}T$	$\mathop \int \limits_{0}^{{298.15}} {C_{\text{V}}}{\text{d}}T$	$\mathop \int \limits_{0}^{{298.15}} \frac{{{C_{\text{P}}}}}{T}{\text{d}}T$	$\mathop \int \limits_{0}^{{298.15}} \frac{{{C_{\text{V}}}}}{T}{\text{d}}T$
	kJ/mol	kJ/mol	J/mol/K	J/mol/K
K_2_O	13.000	14.232	94.140	94.722
CaO	6.749	6.877	38.212	38.839
FeO	9.136	7.533	60.752	44.718
Al_2_O_3_	10.020	10.171	50.950	51.831
SiO_2_	6.916	7.066	41.463	43.733
H_2_O	6.388	3.640	41.632	22.182
hed	28.226	27.109	174.2	159.884
fa	22.489	20.165	151.0	118.871
ann	63.955	57.659	411.4	343.954
Δ_R_-hed	− 1.5	− 1.4	− 7.7	− 11.1
Δ_R_-fa	− 2.7	− 2.0	− 12.0	− 14.3
Δ_R_-ann	− 2.1	− 2.0	− 9.4	− 16.9

Δ_R_-hed, Δ_R_-fa, Δ_R_-ann represents the reaction heat content $\left( {\mathop \int \limits_{0}^{{298.15}} {C_{\text{P}}}{\text{d}}T} \right)$ and reaction entropy $\left( {\mathop \int \limits_{0}^{{298.15}} \frac{{{C_{\text{P}}}}}{T}{\text{d}}T} \right)$ from their oxides, i.e., of the reaction CaO + FeO + 2*SiO_2_ = CaFeSi_2_O_6_ in the case of hedenbergite. The *C*_P_ data were taken from the Janaf-tables (Chase 1998) for the oxides, from Haselton et al. (1987) for hedenbergite, from Robie et al. (1982) for fayalite and from Dachs and Benisek (2015) for annite. The *C*_V_ data were calculated by the DFT method

### Enthalpy

The results of the DFT calculations are summarized and compared to reference values in Table 4. Almost all DFT Δ_f_*H*^298.15^ values are slightly more negative (by a few kJ/mol) than the reference values. The deviation is ~ 7 kJ/mol in average and does not exceed 20 kJ/mol for most minerals. One outlier is pyrope with a deviation of 55.83 kJ/mol. Possible reasons for that are unknown.

**Table 4:** DFT-calculated standard enthalpy and entropy versus reference (Ref) values from the internally consistent data set of Holland and Powell (2011)

Group	Mineral	Δ_f_*H*^298.15^ DFT	Δ_f_*H*^298.15^*-*Reference	Deviation in *H* from Ref	*S* ^298.15^ DFT	*S* ^298.15^ *-*Reference	Deviation in *S* from Ref
		kJ/mol	kJ/mol	kJ/mol	J/mol/K	J/mol/K	J/mol/K
Alumino-silicates	and	$-\hspace{0.17em}2602.21$	$-$ 2588.72	$-\hspace{0.17em}13.49$	89.88	92.70	$-\hspace{0.17em}2.82$
	sill	$-\hspace{0.17em}2598.52$	$-$ 2585.85	$-\hspace{0.17em}12.67$	93.59	95.40	$-\hspace{0.17em}1.81$
	ky	$-\hspace{0.17em}2604.74$	$-\hspace{0.17em}$2593.02	$-\hspace{0.17em}11.72$	82.79	83.50	$-\hspace{0.17em}0.71$
Feldspars	mic	$-\hspace{0.17em}3970.90$	$-\hspace{0.17em}3975.33$	$4.43$	$214.89$	214.30	$0.59$
	ab	$-\hspace{0.17em}3936.48$	$-\hspace{0.17em}3935.49$	$-\hspace{0.17em}0.99$	$206.57$	207.40	$-\hspace{0.17em}0.83$
Olivines	fo	$-\hspace{0.17em}2173.07$	$-\hspace{0.17em}2172.57$	$-\hspace{0.17em}0.49$	$94.34$	95.10	$-\hspace{0.17em}0.76$
	fa	$-\hspace{0.17em}1498.70$	$-\hspace{0.17em}1479.36$	$-\hspace{0.17em}19.29$	$148.67$	151.00	$-\hspace{0.17em}2.33$
Pyroxenes	di	$-\hspace{0.17em}3201.14$	$-\hspace{0.17em}3201.69$	$-\hspace{0.17em}0.55$	$143.15$	142.90	$0.25$
	jd	$-\hspace{0.17em}3027.71$	$-\hspace{0.17em}3025.26$	$-\hspace{0.17em}2.45$	$134.99$	133.50	$1.49$
	hed	$-\hspace{0.17em}2841.59$	$-\hspace{0.17em}2841.92$	$0.33$	$170.75$	175.00	$-\hspace{0.17em}4.25$
Garnets	py	$-\hspace{0.17em}6337.96$	$-\hspace{0.17em}6282.13$	$-\hspace{0.17em}55.83$	$261.19$	269.50	$-\hspace{0.17em}8.31$
	gr	$-\hspace{0.17em}6644.82$	$-\hspace{0.17em}6642.95$	$-\hspace{0.17em}1.88$	$254.23$	255.00	$-\hspace{0.17em}0.77$
OH-containing minerals	ta	$-\hspace{0.17em}5907.43$	$-\hspace{0.17em}5897.17$	$-\hspace{0.17em}10.26$	$266.37$	259.00	$7.37$
	pyp	$-\hspace{0.17em}5656.66$	$-\hspace{0.17em}5640.68$	$-\hspace{0.17em}15.98$	237.99	239.00	$-\hspace{0.17em}1.01$
	phl	$-\hspace{0.17em}6207.55$	$-\hspace{0.17em}6214.95$	$7.40$	$327.18$ ^a^	326.00	$1.18$
	ann	$-\hspace{0.17em}5146.82$	$-\hspace{0.17em}5144.23$	$-\hspace{0.17em}2.59$	$415.50$ ^a^	$418.0$	$-\hspace{0.17em}2.51$
	mu	$-\hspace{0.17em}5981.29$	$-\hspace{0.17em}5976.56$	$-\hspace{0.17em}5.46$	$293.95$ ^a^	292.00	$1.95$
	bru	$-\hspace{0.17em}935.495$	$-\hspace{0.17em}924.664$	$-\hspace{0.17em}10.83$	$63.00$	$63.242$	$-\hspace{0.17em}0.24$
	por	$-\hspace{0.17em}982.433$	$-\hspace{0.17em}986.085$	$3.65$	$79.57$	$83.387$	$-\hspace{0.17em}3.82$
	tr	$-\hspace{0.17em}12313.8$	$-\hspace{0.17em}12304.56$	$-\hspace{0.17em}9.23$	$553.18$	553.00	$0.18$
Oxide	per	$-\hspace{0.17em}1667.80$	$-\hspace{0.17em}1660.63$	$-\hspace{0.17em}7.17$	$92.04$	93.30	$-\hspace{0.17em}1.26$

^a^Configurational entropy, *S*^cfg^ =11.53 J/mol/K added (Dachs and Benisek 2015)

The errors generated by the transformation of *C*_V_ to *C*_P_ can be directly estimated by comparing DFT-calculated Δ_R_*U*^0K^ values with measured Δ_R_*H*^0K^ values. This comparison was undertaken for low microcline as an example and is listed in Table 5. Δ_R_*U*^0K^ is less negative by 4.4 kJ/mol than the measured Δ_R_*H*^0K^ value. On the other hand, the calculated standard enthalpy of this study is also less negative by 4.43 kJ/mol (Table 4) when compared to the tabulated value. This demonstrates that the DFT calculation itself produces a deviation of ~ 4.4 kJ/mol from the experimental standard enthalpy, and not the simplifications made by transforming *C*_V_ to *C*_P_.

**Table 5:** Comparison of the DFT-calculated reaction energy (Δ_R_*U*^0K^) of low microcline with experimentally determined reaction enthalpies (Δ_R_*H*^0K^) at 0 K of the reaction ½ K_2_O + ½ Al_2_O_3_ + 3 SiO_2_ = KAlSi_3_O_8_

	DFT *U*^0K^	Exp *H*^0K^
	kJ/mol	kJ/mol
K_2_O	− 192,377	− 376.171^a^
Al_2_O_3_	− 136,814	− 1685.71^a^
SiO_2_	− 93,949.9	− 917.773^a^
KAlSi_3_O_8_	− 446,666	− 4009.41^b^
Δ_R_*U*^0 K^	− 220.8	
Δ_R_*H*^0 K^		− 225.2

^a^Janaf-table (Chase 1998)

^b^Consisting of Δ_f_*H*^298.15^ = -3975.33 kJ/mol (Holland and Powell 2011) and the heat content between 298.15 and 0 K, ∫*C*_P_ d*T* = − 34.08 kJ/mol (Benisek et al. 2014)

### Entropy

The calculated *S*^298.15^ values are shown in Table 4 and do not deviate more than 4.25 J/mol/K from the reference values except for two outliers. One outlier is again pyrope and the other is talc with deviations of − 8.31 and 7.37 J/mol/K, respectively. The Δ_f_*H*^298.15^ and *S*^298.15^ deviations of pyrope are partly compensated, when the free Gibbs energy (*G*) is considered, but this is not the case for talc, because here the deviation of *H* is negative, but that of *S* is positive.

## Application of the new DFT-calculated *H, S*, and *C*_P_ data

The calculated Δ_f_*H*^298.15^, *S*^298.15^, and *C*_P_ data were tested on various mineral reactions to clarify the impact of their deviations from reference values on phase diagram calculations, thereby taking the values for the molar volume, thermal expansion and bulk modulus from the thermodynamic data set of Holland and Powell (2011). For generating these values by the DFT method, see Sect. “Application of the DFT method to calculate enthalpies and entropies of mineral end members not listed in internally consistent data bases”.

### One-component systems: phase transitions

**Fig. 3:**
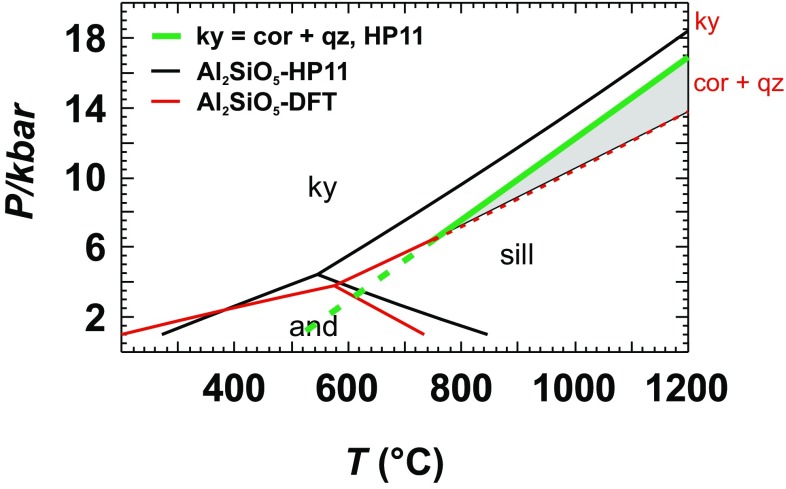
Phase diagram of the Al_2_SiO_5_ system using DFT-calculated standard enthalpy, entropy, and heat capacity values of this study compared to calculations with the Holland and Powell (2011) data set (HP11). The thick curve represents the breakdown reaction of kyanite to corundum + quartz (using the data set of Holland and Powell 2011). The shaded area shows the stability field of corundum + quartz, if DFT-based data are used for the Al_2_SiO_5_ phases. If internally consistent data sets are used instead, the coexistence of quartz + corundum is not possible

Earlier investigations (Benisek and Dachs 2018; Dachs et al. 2018) indicated that the reaction enthalpies (Δ*H*) of phase transitions calculated applying DFT methods are accurate. This can be tested using the new data on the Al_2_SiO_5_ system. Although the new Δ_f_*H*^298.15^ values of andalusite, kyanite, and sillimanite show deviations of more than 10 kJ/mol from reference values, they all have similar deviations. As a consequence, the new Δ*H* values of the phase transitions

11 $${\text{Andalusite }}={\text{ Sillimanite,}}$$

12 $${\text{Kyanite}}\;=\;{\text{Sillimanite,}}$$

and

13 $${\text{Andalusite}}\;=\;{\text{Kyanite,}}$$

do not deviate by more than 1.8 kJ/mol from those of the internally consistent thermodynamic data set of Holland and Powell (2011). To show the impact of these remaining deviations, the new Δ_f_*H*^298.15^, *S*^298.15^, and *C*_P_ data were used to calculate the Al_2_SiO_5_ phase relations and compare it with those derived from the data of Holland and Powell (2011) as shown in Fig. 3. The triple point, where all Al_2_SiO_5_ phases are in equilibrium, is shifted to a slightly higher temperature and lower pressure. The most obvious differences are seen in the slopes of the curves. Some effort was thus undertaken to improve the agreement by testing other functionals than LDA. Although other functionals may lead to better agreement in the absolute values, the delta values of the phase transitions could not be improved. This can be judged from the values listed in Table 6. Using the GGA-PBESOL and PBE0 functionals, the calculated formation energy of kyanite from its oxides shows better agreement with the reference value compared to the LDA functional. Considering the energy difference of the phase transition kyanite = sillimanite, the LDA functional is much better than the other ones. It has been reported that the GGA functional leads overall to an improved agreement with experiment compared to the LDA functional (e.g., Stampfl et al. 2001). Considering differences (Δ*H*, Δ*C*_P_, etc.); however, our experiences are that the LDA functional is slightly superior over the others (e.g., Benisek and Dachs 2012, 2018; Benisek et al. 2018, this study).

**Table 6:** Comparison of the reaction energies for kyanite (ky) and sillimanite (sill) at 0 K (Δ_R_*H*^0K^, Δ_R_*U*^0K^) using different functionals and their reference values (all values in kJ/mol)

	Δ_R_*H*^0K^, Δ_R_*U*^0K^		
	SiO_2_ + Al_2_O_3_ = ky	SiO_2_ + Al_2_O_3_ = sill	ky = sill
Reference	− 5.397^a^	+ 0.199^a^	5.596^a^
LDA	− 17.184	− 12.098	5.086
GGA-PBESOL	− 8.819	− 12.887	− 4.068
B3LYP	+ 3.422	–	–
PBE0	− 2.524	− 19.534	− 17.010

^a^Reference values, taken from the Janaf-tables (Chase 1998) for the oxides, Holland and Powell (2011) for ky and sill, and Robie and Hemingway (1984) for their heat contents between 298.15 and 0 K

However, the differences seen in Fig. 3 have to be discussed from another point of view. Calculations based on internally consistent thermodynamic data bases disagree with petrological findings as described in Harlov and Milke (2002). Although there is petrological evidence that kyanite coexists with quartz and corundum, all available internally consistent data sets predict that this is not possible. At a pressure of 7 kbar for example, kyanite would react to quartz and corundum at a temperature of ~ 800 °C. At this temperature, however, kyanite is not stable with respect to sillimanite (Fig. 3). The kyanite = sillimanite curve must intersect the kyanite = quartz + corundum equilibrium to predict a coexistence of kyanite with quartz + corundum. When using internally consistent data bases, this, however, is not the case for the *P*–*T* range realized in crustal and upper mantle rocks. Extraordinarily, the DFT-based data set of the Al_2_SiO_5_ phases delivers a kyanite = sillimanite curve, which intersects the breakdown curve of kyanite to quartz and corundum (Fig. 3).

### Multi component reactions

The calculations with the Al_2_SiO_5_ phases showed that the DFT method is able to yield accurate thermodynamic data for one-component systems. Now, we will show the impact of the absolute errors in Δ_f_*H*^298.15^ of an Al_2_SiO_5_ phase on a reaction boundary. The DFT-calculated Δ_f_*H*^298.15^ value of kyanite is more negative by 11.72 kJ/mol when compared to the reference value. Since the DFT-derived value is based on the formation from the oxides, this deviation must become effective when calculating the reaction:

14 $${\text{Kyanite}}\;=\;{\text{Quartz}}\;+\;{\text{Corundum.}}$$

At standard conditions, Δ*H* of this reaction is 6.98 kJ/mol (Holland and Powell 2011). The DFT-derived value (18.75 kJ/mol) differs more than 100% from this value. Computing the reaction curve with the DFT-based data shifts the breakdown of kyanite to temperatures as high as 2000 °C, far from being realistic. This reaction was thoroughly investigated by Harlov and Milke (2002) confirming the data from internally consistent data bases. It is, therefore, no doubt that the reaction curve of (14) lies in the *P*–*T* range as shown in Fig. 3 and that the DFT method gave reaction properties for this equilibrium far from being accurate enough. Obviously, the crystal chemistry of the oxides and the Al_2_SiO_5_ phases are too different so that the error inherent in the DFT method is too large, especially in cases where Δ*H* of the investigated reaction is small. In a next step, we now consider reactions that have a larger Δ*H*, so that the DFT errors have a smaller impact on the reaction curves, i.e., dehydration reactions.

### Multi component reactions including H_2_O

**Fig. 4:**
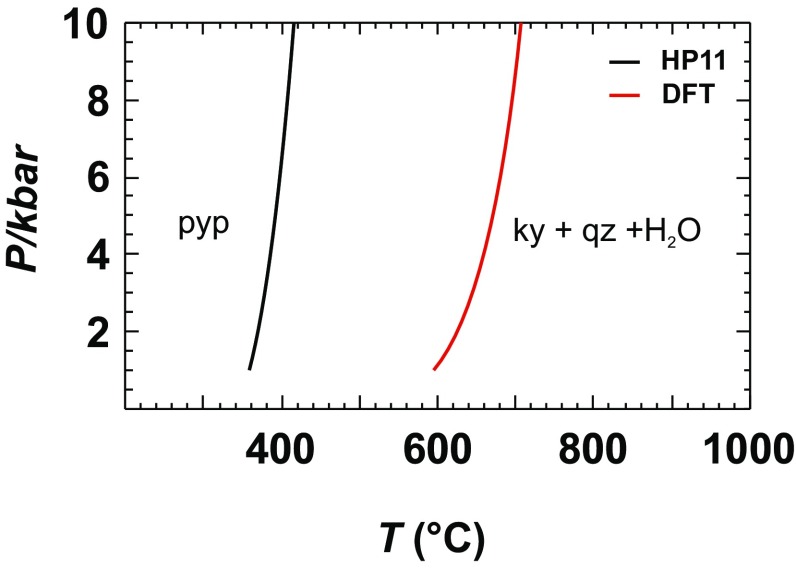
Dehydration reaction of pyrophyllite (pyp) using DFT-calculated standard enthalpy, entropy, and heat capacity values for pyp compared to calculations with the Holland and Powell (2011) data set (HP11) for all phases

To show the full impact of the DFT errors on the phase relations, the thermodynamic data of only one phase are replaced by DFT data for the reactions to follow. If the data of two or more phases would be replaced, than the errors become smaller in most cases because of their covariance, i.e., most of the enthalpic DFT data deviate negatively from the reference values and, thus, errors would partly be compensated. The results of the following reaction,

15 $${\text{Pyrophyllite}}\;=\;{\text{Kyanite}}\;+\;{\text{Quartz}}\;+\;{{\text{H}}_{\text{2}}}{\text{O,}}$$

are shown in Fig. 4. One curve is calculated with Δ_f_*H*^298.15^, *S*^298.15^, and *C*_P_ data for pyrophyllite as determined by the DFT method. This reaction has a Δ*H* of ca. 73 kJ/mol. The DFT-based Δ_f_*H*^298.15^ deviates by 15.98 kJ/mol from the reference value (Table 4) and this causes a shift of ca. 200 °C in the calculated *P−T* position (Fig. 4), a value which is still too large.

**Fig. 5:**
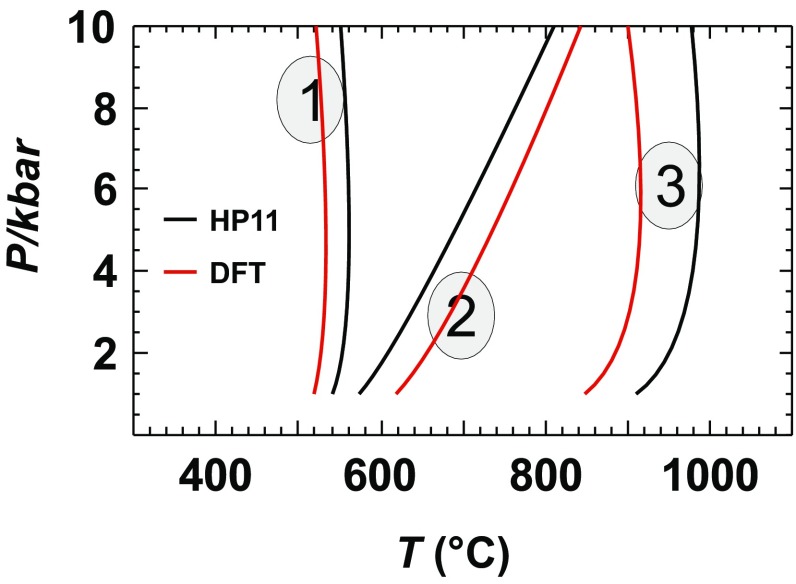
Dehydration reaction of micas: (1) annite + kyanite + 2 quartz = almandine + sanidine + H_2_O; (2) muscovite + quartz = sillimanite + sanidine + H_2_O; (3) phlogopite + sillimanite + 2 quartz = pyrope + sanidine + H_2_O. Calculations were done using DFT-derived standard enthalpy, entropy, and heat capacity values of annite, muscovite, and phlogopite. They are compared to calculations with the Holland and Powell (2011) data set (HP11) for all phases

The reactions,

16 $${\text{Annite}}\;+\;{\text{Kyanite}}\;+\;{\text{2 Quartz}}\;=\;{\text{Almandine}}\;+\;{\text{Sanidine}}\;+\;{{\text{H}}_{\text{2}}}{\text{O,}}$$

17 $${\text{Muscovite}}\;+\;{\text{Quartz}}\;=\;{\text{Sillimanite}}\;+\;{\text{Sanidine}}\;+\;{{\text{H}}_{\text{2}}}{\text{O,}}$$

18 $${\text{Phlogopite}}\;+\;{\text{Sillimanite}}\;+\;{\text{2 Quartz}}\;=\;{\text{Pyrope}}\;+\;{\text{Sanidine}}\;+\;{{\text{H}}_{\text{2}}}{\text{O,}}$$

were then investigated and the results are shown in Fig. 5. The Δ*H*’s of these reactions are larger than 100 kJ/mol and the DFT-based enthalpies deviate less than 10 kJ/mol from reference values (Table 4). As a consequence, the effect of the DFT errors on the reaction temperatures is small (Fig. 5).

From the errors of DFT-computed Δ_f_*H*^298.15^ and *S*^298.15^ that of the entropy has a smaller impact on the phase relations. This can be shown for sillimanite as an example. At a temperature of 1000 K, the error in *S*^298.15^ produces an error in the Gibbs free energy (*G*) of − 1.81 kJ/mol, whereas for that of the enthalpy an error in *G* of − 12.67 kJ/mol (see Table 4).

### Application of the DFT method to calculate enthalpies and entropies of mineral end members not listed in internally consistent data bases

To present DFT data for a less known end member, we chose ordered Al-buffonite (CaTi_1/2_Mg_1/2_AlSiO_6_), because it is an important pyroxene end member in calcium–aluminium-rich inclusions (CAIs) of primitive meteorites and their thermodynamic data were only investigated by a few studies (e.g., Sack and Ghiorso 1994, 2017). The DFT-based thermodynamic standard data (Δ_f_*H*^298.15^, *S*^298.15^) are listed in Table 7, where they are compared to those of Sack and Ghiorso (2017) showing excellent agreement supporting their work. If a value for the volume, *V*^298.15, 1 bar^, is required, it can be calculated from the DFT-based volume (*V*^0K^) in a similar manner to that described above for *C*_*P*_, i.e., by evaluating a relative mean difference between *V*^298.15, 1 bar^ and *V*^0K^ of the well-known pyroxene end members, diopside, jadeite, and hedenbergite:

**Table 7:** Thermodynamic data calculated by the DFT method and compared to literature data for ordered Al-buffonite (CaTi_1/2_Mg_1/2_AlSiO_6_)

	Δ_f_*H*^298.15^ (kJ/mol)	*S* ^298.15^ (J/mol/K)
This study	− 3283.64	143.019
Sack and Ghiorso (2017)	− 3283.83	143.745

19 $$\Delta V\;=\;\left( {{V^{{\text{298}}.{\text{15}},{\text{ 1 bar}}}} - {V^{0{\text{K}}}}} \right)/{V^{0{\text{K}}}}.$$

The volume at 298.15 K of the unknown pyroxene is then given by

20 $${V^{{\text{298}}.{\text{15}},{\text{ 1 bar}}}}\;=\;{V^{0{\text{K}}}}\left( {{\text{1 }}+\Delta V} \right).$$

Values for thermal expansion and for the bulk modulus may be derived from averaging the values of the well-known phases of this particular mineral group. If, however, these values have to be determined accurately, i.e., for HP–HT phases, then the quasi harmonic approximation should be used instead (see, e.g., Belmonte 2017).

## Comparison with estimation methods

**Fig. 6:**
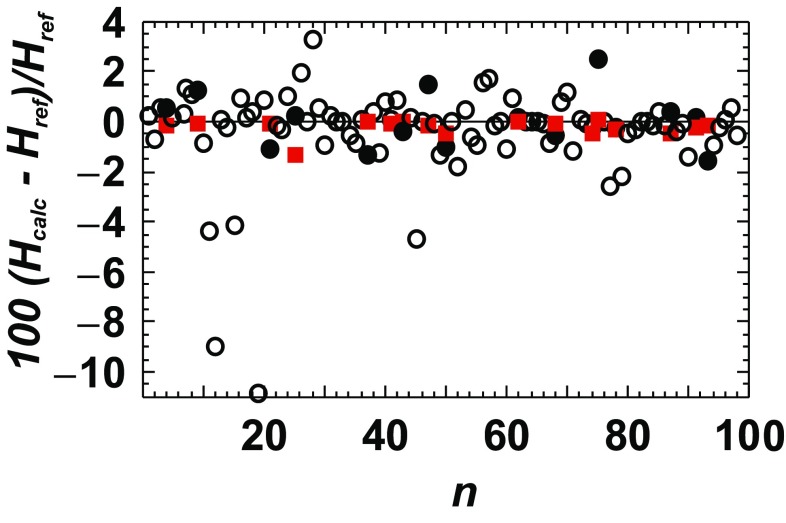
DFT versus estimation method of van Hinsburg et al. (2005). The relative deviation of the calculated standard enthalpy ((*H*_calc_−*H*_ref_)/*H*_ref_) from reference values (Holland and Powell 2011) are plotted for the minerals investigated in van Hinsburg et al. (2005). Squares represent the DFT data of this study, which are compared to the estimated values (filled circles). Open circles represent estimated values for minerals not investigated by the DFT method

The relative deviations of DFT-calculated enthalpies from reference values (*H*_calc_−*H*_ref_)/*H*_ref_ are compared in Fig. 6 to those resulting from an enthalpy estimation method (van Hinsberg et al. 2005). Clearly, the DFT method yields by far more accurate results. The DFT-calculated Δ_f_*H*^298.15^ values deviate by not more than 1.3% from the reference values, whereas those derived from the estimation method show deviations of more than 10%. For almost all investigated phases, the DFT method is more accurate. A similar picture can be drawn for *S*^298.15^.

## Conclusions

DFT-based thermodynamic data are suitable to calculate reaction curves of phase transitions as well as of reactions, whose Δ*H*’s are large (> 100 kJ/mol). For other reactions, this method does not yield accurate enough thermodynamic data. DFT-calculated energies have uncertainties in the order of 10 kJ/mol, slightly larger than those of calorimetric methods. The DFT data are, however, far better than data generated by estimation methods. The optimal approach will be to use DFT-calculated thermodynamic data together with those from other sources (calorimetry, phase equilibrium experiments) to develop internally consistent databases.
